# Supplementary material for: A 30-Year Experience in Fragile X Syndrome Molecular Diagnosis from a Laboratory in Thailand
Source: Int J Mol Sci. 2025 Aug 1;26(15):7418. doi: 10.3390/ijms26157418 (PMC12347110; doi:10.3390/ijms26157418)
Supplement: Supplementary file 1 [file ijms-26-07418-s001.zip › ijms-3755978-supplementary.pdf]

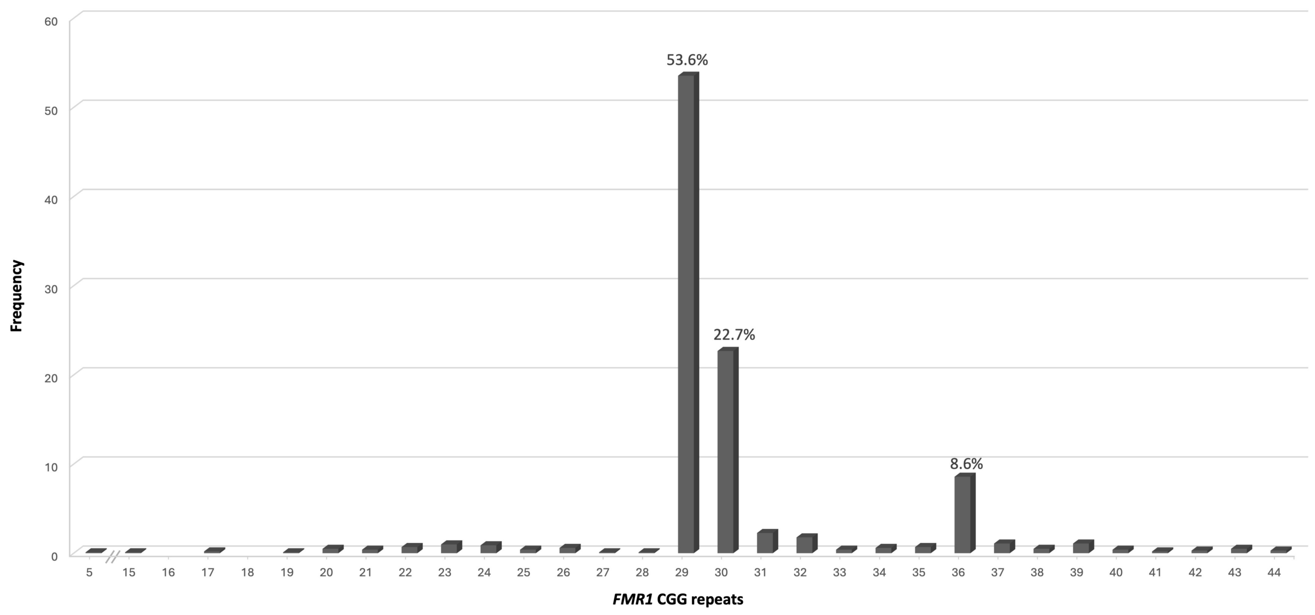

Figure S1. Distribution of normal CGG repeats in the *FMR1* gene among 1,014 male patients, which includes 93 males from a cohort of individuals with intellectual disability from the Child psychiatric clinic/Northern Thailand, and 921 males from a cohort of children undergoing routine fragile X syndrome testing at Songklanagarind Hospital, representing all regions of Thailand. The CGG repeat numbers were determined based on the results from fluorescent PCR followed by capillary electrophoresis. The most common alleles are 29 and 30 CGG repeats, followed by 36 CGG repeats.

Table S1. Full mutation expansions and AGG interruptions on *FMR1* maternal transmission

Maternal repeat size	No. AGGs	Number of children with full mutation expansion
70-79	0 AGG	3[b]
	1 AGG	1
80-89	0 AGG	4
	1 AGG	2
90-99	0 AGG	2
	1 AGG	2
100-109	0 AGG	7
	1 AGG	2[b]
110-119	0 AGG	2
	1 AGG	2
120-129	0 AGG	-
	1 AGG	-
130-139	0 AGG	-
	1 AGG	-
140-149	0 AGG	1[a]
	1 AGG	-
150-199	0 AGG	4[b]
	1 AGG	-
>200	0 AGG	2[b]
	1 AGG	-
**Total**		**34[c]**

Please note that, in cases of patients with the full mutation in this cohort, no mother was found to carry alleles with < 70 CGG repeats.

Dash (-) indicates no mother carrier CGG repeats in this range.

[a] A mother carrier has 37/~140/>200 CGG repeats and has one child with a full mutation and another child with a mosaic premutation (~101 bp)/313 bp deletion [1].

[b] Mother in this group had more than one child with a full mutation.

[c] Among 34 patients with full mutations whose mothers were tested for AGG interruptions, TP-PCR analysis revealed 25 full mutation transmissions from maternal alleles with no AGG interruptions and nine transmissions from premutation alleles with one AGG interruption.

Table S2. Frequency of fragile X syndrome in high-risk groups worldwide using molecular testing (sample sizes ranging from 100 to >1000).

**Location**	**Subjects**	**No. of patients**	**Methods**	**FXS frequency (FM and mosaic FM)**	**Reference**
**Asia**					
**Southeast Asia**					
Indonesia	Patients with DD	262	PCR with gel<br>SB	1.5% (4/262)<br>M: 1.9% (4/206)	Faradz et al. 1999 [2]
Indonesia	Patient with ID, ASD, FHx of FXS	176<br>M = 92<br>F = 84	PCR with gel or CE<br>TP-PCR<br>SB	4.0% (7/176)<br>M: 1.1% (1/92)<br>F: 7.1% (6/84)	Winarni et al. 2012 [3]
Malaysia	Patients with DD	2,184<br>M = 2,057<br>F = 127	PCR with gel or CE<br>MS-PCR<br>FragilEase kit	3.5% (76/2,184)<br>M: 3.5% (73/2,057)<br>F: 2.4% (3/127)	Ali et al. 2017 [4]
Singapore	Males with LD	M = 255	PCR with gel<br>SB	M: 2.4% (6/255)	Tan et al. 2000 [5]
Thailand	Patients with DD, ID, ASD	1,480<br>M = 1,390<br>F = 90	PCR with gel or CE<br>MS-PCR<br>TP-PCR<br>msTP-PCR<br>SB	7% (103/1,480)<br>M: 7.2% (100/1,390)<br>F: 3.3% (3/90)	This study
**East Asia**					
China	Patients with ID	1,127	PCR with gel<br>SB	2.8% (32/1,127)[a]	Zhong et al. 1999 [6]
China	Patients with ID	324<br>M = 243<br>F = 81	PCR with gel<br>SB	0.6% (2/324)<br>M: 0.4% (1/243)<br>F: 1.2% (1/81)	Pang et al. 1999 [7]
China	Patients with ID, DD	540<br>M = 453<br>F = 87	PCR with gel<br>TP-PCR	0.9% (5/540)<br>M: 1.1% (5/453)<br>F: 0% (0/87)	Chen et al. 2015 [8]
Japan	Patients with ID	425<br>M = 296<br>F = 129	PCR with gel<br>SB	2.1% (9/425)<br>M: 2.4% (7/296)<br>F: 1.6% (2/129)	Hofstee et al. 1994 [9]
Japan	Males with ID	M = 256	PCR with gel<br>SB	M: 0.8% (2/256)	Nanba et al. 1995 [10]
Japan	Patients with ASD	109<br>M = 102<br>F = 7	PCR with CE<br>SB	0% (0/109)[a]	Otsuka et al. 2010 [11]
Korea	Patients with ID, LD, ASD	101<br>M = 78<br>F = 23	PCR with gel<br>SB	1% (1/101)<br>M: 1.3% (1/78)<br>F: 0% (0/23)	Kwon et al. 2001 [12]
Taiwan	Patients with ID	471<br>M = 311<br>F = 160	PCR with gel<br>SB	2.1% (10/471)<br>M: 2.9% (9/311)<br>F: 0.6% (1/160)	Tzeng et al. 2001 [13]
Taiwan	Patients with ID	217<br>M = 148<br>F = 69	PCR with gel<br>SB	1.4% (3/217)<br>M: 1.4% (2/148)<br>F: 1.4% (1/69)	Yen et al. 2008 [14]
**South Asia**					
India	Patients with ID	130<br>M = 93<br>F = 37	PCR with gel<br>SB	7.7% (10/130)<br>M: 9.7% (9/93)<br>F: 2.7% (1/37)	Sharma et al. 2001 [15]

Table S2. (continued)

Location	Subjects	No. of patients	Methods	FXS frequency<br>(FM and mosaic FM)	Reference
India	Patients with ID	146<br>M = 118<br>F = 28	PCR with gel<br>SB	2.1% (3/146)<br>M: 2.5% (3/118)<br>F: 0% (0/28)	Pandey et al. 2002 [16]
Pakistan	Patients with ID	333<br>M = 229<br>F = 104	PCR with gel<br>MS-PCR<br>SB	4.8% (16/333)<br>M: 6.6% (15/229)<br>F: 1% (1/104)	Fatima et al. 2014 [17]
Pakistan	Patients with ID	395<br>M = 287<br>F = 108	PCR with gel<br>MS-PCR<br>SB	3.3% (13/395)<br>M: 3.5% (10/287)<br>F: 2.8% (3/108)	Kanwal et al. 2015 [18]
Sri Lanka	Patients with ID, DD, ASD, ADHD	850<br>M = 540<br>F = 310	TP-PCR<br>MS-PCR<br>SB	1.3% (11/850)<br>M: 2% (11/540)<br>F: 0% (0/310)	Chandrasekara et al. 2017 [19]
**Middle East**					
Iran	Patients with ID[b]	508 (Proband)<br>M = 428<br>F = 80	PCR with gel<br>SB	6.3% (32/508)<br>M: 7.2% (31/428)<br>F: 1.3% (1/80)	Pouya et al. 2009 [20]
Kuwait	Males with ID	M = 182	Cytogenetics<br>PCR with gel	M: 11% (20/182)	Bastaki et al. 2004 [21]
**Europe**					
Croatia	Patients with ID	114<br>M = 73<br>F = 41	PCR with gel<br>PCR-Chemi	2.6% (3/114)<br>M: 2.7% (2/73)<br>F: 2.4% (1/41)	Hećimović et al. 2002 [22]
Estonia	Patients with ID	516<br>M = 448<br>F = 68	PCR with CE<br>SB	2.7% (14/516)<br>M: 3.1% (14/448)<br>F: 0% (0/68)	Puusepp et al. 2008 [23]
France	Patients with ID	574<br>M = 403<br>F = 171	PCR<br>SB	1.9% (11/574)<br>M: 2.5% (10/403)<br>F: 0.6% (1/171)	Gérard et al. 1997 [24]
France	Patients with ID	20,816<br>M = 14,867<br>F = 5,949	PCR<br>SB	2.3% (477/20,816)<br>M: 2.8% (417/14,867)<br>F: 1% (60/5,949)	Biancalana et al. 2004 [25]
Greece	Patients with ID	1,755<br>M = 1,426<br>F = 329	PCR with CE<br>SB	3.5% (61/1,755)<br>M: 4% (57/1,426)<br>F: 1.2% (4/329)	Sofocleous et al. 2008 [26]
Italy	Patients with ID, DD, ASD	2,832<br>2,750 patients with ID, DD<br>82 patients with ASD	PCR with CE<br>TP-PCR<br>SB	2.9% (82/2,832)[a]<br>ID, DD: 2.9% (81/2,750)<br>ASD: 1.2% (1/82)	Esposito et al. 2013 [27]
Latvia	Males with ID	M = 374	PCR with CE<br>SB	M: 2.7% (10/374)	Daneberga et al. 2011 [28]
Netherland	Patients with ID	236<br>M = 197<br>F = 39	PCR with gel<br>SB	4.2% (10/236)[a]	van den Ouweland et al. 1994 [29]
Netherland	Patients with ID	1,531<br>M = 870<br>F = 661	PCR with gel<br>SB	0.7% (11/1,531)<br>M: 1% (9/870)<br>F: 0.3% (2/661)	de Vries et al. 1997 [30]
Spain	Patients with ID	222<br>M = 182<br>F = 40	SB	5% (11/222)<br>M: 6% (11/182)<br>F: 0% (0/40)	Milà et al. 1997 [31]

Table S2. (continued)

Location	Subjects	No. of patients	Methods	FXS frequency (FM and mosaic FM)	Reference
Spain	Subjects with ID	392<br>M = 277<br>F = 115	PCR with gel<br>SB<br>Sequencing	5.6% (22/392)<br>M: 6.5% (18/277)<br>F: 3.5% (4/115)	Millán et al. 1999 [32]
Turkey	Patients with ID, DD	179<br>M = 166<br>F = 13	PCR with gel<br>SB	2.8% (5/179)<br>M: 3% (5/166)<br>F: 0% (0/13)	Tunçbilek et al. 1999 [33]
UK	Patients with LD	154<br>M = 103<br>F = 51	Cytogenetics<br>SB	2.6% (4/154)<br>M: 3.9% (4/103)<br>F: 0% (0/51)	Slaney et al. 1995 [34]
UK	Males with LD	M = 3,732	PCR<br>SB	M: 0.5% (20/3,732)	Youings et al. 2000 [35]
**North America**					
USA (Georgia)	Children in special education classes	3,532<br>M = 2,471<br>F =1,061	PCR<br>SB	0.2% (7/3,532)<br>M: 0.3% (7/2,471)<br>F: 0% (0/1061)	Crawford et al. 2002 [36]
USA	Patients with ASD	316	PCR<br>SB	1.9% (6/316)[a]	Reddy et al. 2005 [37]
USA	Males with mental dysfunction	M = 59,707	PCR with gel or CE<br>SB	M: 1.4% (862/59,707)	Strom et al. 2007 [38]
USA	Patients with DD, ASD	599<br>M = 486<br>F = 113	PCR with CE<br>TP-PCR	0.7% (4/599)<br>M: 0.6% (3/486)<br>F: 0.9% (1/113)	Tassone et al. 2013 [39]
**South America**					
Chile	Patients with ID and clinical features of FXS	2,202	PCR with gel or CE<br>SB	8.2 % (181/2,202)[a]	Santa María L et al. 2016 [40]
Ecuador	Males with ID, ASD, language delay, FHx of FXS	M = 247	PCR with gel	M: 8.9% (22/247)	Pozo-Palacios J et al. 2021 [41]
**Australia**					
Australia (Tasmania)	Patients with special educational needs (ID, LD, ASD, ADHD)	1,248	PCR with gel<br>SB	0% (0/1,248)[a]	Mitchell et al. 2004 [42]
**Africa**					
Egypt	Males with ID, LD	M = 400	PCR with gel	M: 4.8% (19/400)	Meguid et al. 2007 [43]
South Africa	Males with ID	148	SB	M: 6.1% (9/148)	Goldman et al. 1998 [44]
South Africa	Patients with ID	2,239<br>M = 1,961<br>F = 278	PCR with CE<br>SB	5.7 % (128/2,239)<br>M: 6% (117/1,961)<br>F: 4% (11/278)	Essop et al. 2013 [45]
**Systematic review/ meta-analysis**					
Systematic review and meta-analysis	Patients with ID[c]	7,475	PCR<br>SB	2.4% (178/7,475)[a]	Hunter et al. 2014 [46]

ID, intellectual disability; ASD, autism spectrum disorder; DD, developmental delay; LD, learning disability; MS-PCR, methylation-specific PCR; PCR with gel, PCR followed by gel electrophoresis; PCR with CE, PCR followed

by capillary electrophoresis; PCR-Chemi, PCR followed by chemiluminescent detection; TP-PCR, triplet repeat-primed PCR; SB, Southern blot analysis; M, male; F, female; FM, full mutation (> 200 CGG repeats)

[a] Based on original research, the data does not provide frequencies separated by male and female; [b] Consanguineous and non-consanguineous parents; [c] Searching primary publication in PubMed, Embase, and the Cochrane library

References

1. Hnoonual, A.; Plong-On, O.; Worachotekamjorn, J.; Charalsawadi, C.; Limprasert, P. Clinical and Molecular Characteristics of FMR1 Microdeletion in Patient with Fragile X Syndrome and Review of the Literature. *Clin Chim Acta* **2024**, *553*, 117728, doi:10.1016/j.cca.2023.117728.
2. Faradz, S.M.; Buckley, M.; Lam-Po-Tang, null; Leigh, D.; Holden, J.J. Molecular Screening for Fragile X Syndrome among Indonesian Children with Developmental Disability. *Am J Med Genet* **1999**, *83*, 350–351.
3. Winarni, T.I.; Utari, A.; Mundhofir, F.E.P.; Tong, T.; Durbin-Johnson, B.; Faradz, S.M.H.; Tassone, F. Identification of Expanded Alleles of the FMR1 Gene among High-Risk Population in Indonesia by Using Blood Spot Screening. *Genet Test Mol Biomarkers* **2012**, *16*, 162–166, doi:10.1089/gtmb.2011.0089.
4. Ali, E.Z.; Yakob, Y.; Md Desa, N.; Ishak, T.; Zakaria, Z.; Ngu, L.K.; Keng, W.T. Molecular Analysis of Fragile X Syndrome (FXS) among Malaysian Patients with Developmental Disability. *Malays J Pathol* **2017**, *39*, 99–106.
5. Tan, B.S.; Law, H.Y.; Zhao, Y.; Yoon, C.S.; Ng, I.S. DNA Testing for Fragile X Syndrome in 255 Males from Special Schools in Singapore. *Ann Acad Med Singap* **2000**, *29*, 207–212.
6. Zhong, N.; Ju, W.; Xu, W.; Ye, L.; Shen, Y.; Wu, G.; Chen, S.H.; Jin, R.; Hu, X.F.; Yang, A.; et al. Frequency of the Fragile X Syndrome in Chinese Mentally Retarded Populations Is Similar to That in Caucasians. *Am J Med Genet* **1999**, *84*, 191–194, doi:10.1002/(sici)1096-8628(19990528)84:3<191::aid-ajmg3>3.0.co;2-8.
7. Pang, C.P.; Poon, P.M.; Chen, Q.L.; Lai, K.Y.; Yin, C.H.; Zhao, Z.; Zhong, N.; Lau, C.H.; Lam, S.T.; Wong, C.K.; et al. Trinucleotide CGG Repeat in the FMR1 Gene in Chinese Mentally Retarded Patients. *Am J Med Genet* **1999**, *84*, 179–183.
8. Chen, X.; Wang, J.; Xie, H.; Zhou, W.; Wu, Y.; Wang, J.; Qin, J.; Guo, J.; Gu, Q.; Zhang, X.; et al. Fragile X Syndrome Screening in Chinese Children with Unknown Intellectual Developmental Disorder. *BMC Pediatr* **2015**, *15*, 77, doi:10.1186/s12887-015-0394-8.
9. Hofstee, Y.; Arinami, T.; Hamaguchi, H. Comparison between the Cytogenetic Test for Fragile X and the Molecular Analysis of the FMR-1 Gene in Japanese Mentally Retarded Individuals. *Am J Med Genet* **1994**, *51*, 466–470, doi:10.1002/ajmg.1320510434.
10. Nanba, E.; Kohno, Y.; Matsuda, A.; Yano, M.; Sato, C.; Hashimoto, K.; Koeda, T.; Yoshino, K.; Kimura, M.; Maeoka, Y.; et al. Non-Radioactive DNA Diagnosis for the

Fragile X Syndrome in Mentally Retarded Japanese Males. *Brain and Development* **1995**, *17*, 317–321, doi:10.1016/0387-7604(95)00031-6.
11. Otsuka, S.; Sakamoto, Y.; Siomi, H.; Itakura, M.; Yamamoto, K.; Matumoto, H.; Sasaki, T.; Kato, N.; Nanba, E. Fragile X Carrier Screening and FMR1 Allele Distribution in the Japanese Population. *Brain Dev* **2010**, *32*, 110–114, doi:10.1016/j.braindev.2008.12.015.
12. Kwon, S.H.; Lee, K.S.; Hyun, M.C.; Song, K.E.; Kim, J.K. Molecular Screening for Fragile X Syndrome in Mentally Handicapped Children in Korea. *J Korean Med Sci* **2001**, *16*, 271–275, doi:10.3346/jkms.2001.16.3.271.
13. Tzeng, C.C.; Lin, S.J.; Chen, Y.J.; Kuo, P.L.; Jong, Y.J.; Tsai, L.P.; Chen, R.M. An Effective Strategy of Using Molecular Testing to Screen Mentally Retarded Individuals for Fragile X Syndrome. *Diagn Mol Pathol* **2001**, *10*, 34–40, doi:10.1097/00019606-200103000-00006.
14. Yen, J.-H.; Chen, W.-C.; Tzeng, C.-C.; Fang, J.-S.; Chu, S.-Y. Molecular Screening of Fragile X Syndrome in Children with Mental Retardation in Hualien. *Tzu Chi Medical Journal* **2008**, *20*, 309–313, doi:10.1016/S1016-3190(08)60056-7.
15. Sharma, D.; Gupta, M.; Thelma, B.K. Expansion Mutation Frequency and CGG/GCC Repeat Polymorphism in FMR1 and FMR2 Genes in an Indian Population. *Genet. Epidemiol.* **2001**, *20*, 129–144, doi:10.1002/1098-2272(200101)20:1<129::AID-GEPI11>3.0.CO;2-2.
16. Pandey, U.B.; Phadke, S.; Mittal, B. Molecular Screening of FRAXA and FRAXE in Indian Patients with Unexplained Mental Retardation. *Genet Test* **2002**, *6*, 335–339, doi:10.1089/10906570260471903.
17. Fatima, T.; Zaidi, S.A.H.; Sarfraz, N.; Perween, S.; Khurshid, F.; Imtiaz, F. Frequency of FMR1 Gene Mutation and CGG Repeat Polymorphism in Intellectually Disabled Children in Pakistan. *Am. J. Med. Genet. A* **2014**, *164A*, 1151–1161, doi:10.1002/ajmg.a.36423.
18. Kanwal, M.; Alyas, S.; Afzal, M.; Mansoor, A.; Abbasi, R.; Tassone, F.; Malik, S.; Mazhar, K. Molecular Diagnosis of Fragile X Syndrome in Subjects with Intellectual Disability of Unknown Origin: Implications of Its Prevalence in Regional Pakistan. *PLoS One* **2015**, *10*, e0122213, doi:10.1371/journal.pone.0122213.
19. Chandrasekara, B.; Wijesundera, S.; Chong, S.S.; Perera, H.N. Prevalence of Fragile X Syndrome among Children Receiving Special Education and Carrier States in First Degree Relatives. *Ceylon Med J* **2017**, *62*, 92–96, doi:10.4038/cmj.v62i2.8473.

20. Pouya, A.R.; Abedini, S.S.; Mansoorian, N.; Behjati, F.; Nikzat, N.; Mohseni, M.; Nieh, S.E.; Abbasi Moheb, L.; Darvish, H.; Monajemi, G.B.; et al. Fragile X Syndrome Screening of Families with Consanguineous and Non-Consanguineous Parents in the Iranian Population. *Eur J Med Genet* **2009**, *52*, 170–173, doi:10.1016/j.ejmg.2009.03.014.
21. Bastaki, L.A.; Hegazy, F.; Al-Heneidi, M.M.; Turki, N.; Azab, A.S.; Naguib, K.K. Fragile X Syndrome: A Clinico-Genetic Study of Mentally Retarded Patients in Kuwait. *East Mediterr Health J* **2004**, *10*, 116–124.
22. Hećimović, S.; Tarnik, I.P.; Barić, I.; Cakarun, Z.; Pavelić, K. Screening for Fragile X Syndrome: Results from a School for Mentally Retarded Children. *Acta Paediatr.* **2002**, *91*, 535–539, doi:10.1080/080352502753711650.
23. Puusepp, H.; Kahre, T.; Sibul, H.; Soo, V.; Lind, I.; Raukas, E.; Ounap, K. Prevalence of the Fragile X Syndrome among Estonian Mentally Retarded and the Entire Children's Population. *J Child Neurol* **2008**, *23*, 1400–1405, doi:10.1177/0883073808319071.
24. Gérard, B.; Le Heuzey, M.F.; Brunie, G.; Lewine, P.; Saiag, M.C.; Cacheux, V.; Da Silva, F.; Dugas, M.; Mouren-Simeoni, M.C.; Elion, J.; et al. Systematic Screening for Fragile X Syndrome in a Cohort of 574 Mentally Retarded Children. *Ann Genet* **1997**, *40*, 139–144.
25. Biancalana, V.; Beldjord, C.; Taillandier, A.; Szpiro-Tapia, S.; Cusin, V.; Gerson, F.; Philippe, C.; Mandel, J.-L. Five Years of Molecular Diagnosis of Fragile X Syndrome (1997-2001): A Collaborative Study Reporting 95% of the Activity in France. *Am J Med Genet A* **2004**, *129A*, 218–224, doi:10.1002/ajmg.a.30237.
26. Sofocleous, C.; Kitsiou, S.; Fryssira, H.; Kolialexi, A.; Kalaitzidaki, M.; Roma, E.; Tsangaris, G.T.; Chistofidou, C.; Metaxotou, C.; Kanavakis, E.; et al. 10 Years' Experience in Fragile X Testing among Mentally Retarded Individuals in Greece: A Molecular and Epidemiological Approach. *In Vivo* **2008**, *22*, 451–455.
27. Esposito, G.; Ruggiero, R.; Savarese, G.; Savarese, M.; Tremolaterra, M.R.; Salvatore, F.; Carsana, A. A 15-Year Case-Mix Experience for Fragile X Syndrome Molecular Diagnosis and Comparison between Conventional and Alternative Techniques Leading to a Novel Diagnostic Procedure. *Clin Chim Acta* **2013**, *417*, 85–89, doi:10.1016/j.cca.2012.12.021.
28. Daneberga, Z.; Krūmiņa, Z.; Lāce, B.; Bauze, D.; Lugovska, R. The Fragile X Syndrome: 13 Years of Experience. *Proceedings of the Latvian Academy of Sciences. Section B. Natural, Exact, and Applied Sciences.* **2011**, *65*, 67–72.
29. van den Ouweland, A.M.; de Vries, B.B.; Bakker, P.L.; Deelen, W.H.; de Graaff, E.; van Hemel, J.O.; Oostra, B.A.; Niermeijer, M.F.; Halley, D.J. DNA Diagnosis of the Fragile X

Syndrome in a Series of 236 Mentally Retarded Subjects and Evidence for a Reversal of Mutation in the FMR-1 Gene. *Am J Med Genet* **1994**, *51*, 482–485, doi:10.1002/ajmg.1320510437.

30. de Vries, B.B.; van den Ouweland, A.M.; Mohkamsing, S.; Duivenvoorden, H.J.; Mol, E.; Gelsema, K.; van Rijn, M.; Halley, D.J.; Sandkuijl, L.A.; Oostra, B.A.; et al. Screening and Diagnosis for the Fragile X Syndrome among the Mentally Retarded: An Epidemiological and Psychological Survey. Collaborative Fragile X Study Group. *Am J Hum Genet* **1997**, *61*, 660–667, doi:10.1086/515496.
31. Milà, M.; Sànchez, A.; Badenas, C.; Brun, C.; Jiménez, D.; Villa, M.P.; Castellví-Bel, S.; Estivill, X. Screening for FMR1 and FMR2 Mutations in 222 Individuals from Spanish Special Schools: Identification of a Case of FRAXE-Associated Mental Retardation. *Hum Genet* **1997**, *100*, 503–507, doi:10.1007/s004390050542.
32. Millán, J.M.; Martínez, F.; Cadroy, A.; Gandía, J.; Casquero, M.; Beneyto, M.; Badía, L.; Prieto, F. Screening for FMR1 Mutations among the Mentally Retarded: Prevalence of the Fragile X Syndrome in Spain. *Clin. Gen.* **1999**, *56*, 98–99, doi:10.1034/j.1399-0004.1999.560116.x.
33. Tunçbilek, E.; Alikasifoğlu, M.; Boduroğlu, K.; Aktas, D.; Anar, B. Frequency of Fragile X Syndrome among Turkish Patients with Mental Retardation of Unknown Etiology. *Am J Med Genet* **1999**, *84*, 202–203, doi:10.1002/(sici)1096-8628(19990528)84:3<202::aid-ajmg6>3.0.co;2-o.
34. Slaney, S.F.; Wilkie, A.O.; Hirst, M.C.; Charlton, R.; McKinley, M.; Pointon, J.; Christodoulou, Z.; Huson, S.M.; Davies, K.E. DNA Testing for Fragile X Syndrome in Schools for Learning Difficulties. *Arch Dis Child* **1995**, *72*, 33–37, doi:10.1136/adc.72.1.33.
35. Youings, S.A.; Murray, A.; Dennis, N.; Ennis, S.; Lewis, C.; McKechnie, N.; Pound, M.; Sharrock, A.; Jacobs, P. FRAXA and FRAXE: The Results of a Five Year Survey. *J Med Genet* **2000**, *37*, 415–421, doi:10.1136/jmg.37.6.415.
36. Crawford, D.C.; Meadows, K.L.; Newman, J.L.; Taft, L.F.; Scott, E.; Leslie, M.; Shubek, L.; Holmgreen, P.; Yeargin-Allsopp, M.; Boyle, C.; et al. Prevalence of the Fragile X Syndrome in African-Americans. *Am J Med Genet* **2002**, *110*, 226–233, doi:10.1002/ajmg.10427.
37. Reddy, K.S. Cytogenetic Abnormalities and Fragile-X Syndrome in Autism Spectrum Disorder. *BMC Med Genet* **2005**, *6*, 3, doi:10.1186/1471-2350-6-3.

38. Strom, C.M.; Crossley, B.; Redman, J.B.; Buller, A.; Quan, F.; Peng, M.; McGinnis, M.; Fenwick, R.G.; Sun, W. Molecular Testing for Fragile X Syndrome: Lessons Learned from 119,232 Tests Performed in a Clinical Laboratory. *Genet Med* **2007**, *9*, 46–51, doi:10.1097/gim.0b013e31802d833c.
39. Tassone, F.; Choudhary, N.S.; Tassone, F.; Durbin-Johnson, B.; Hansen, R.; Hertz-Picciotto, I.; Pessah, I. Identification of Expanded Alleles of the FMR1 Gene in the CHildhood Autism Risks from Genes and Environment (CHARGE) Study. *J Autism Dev Disord* **2013**, *43*, 530–539, doi:10.1007/s10803-012-1580-2.
40. Santa María, L.; Aliaga, S.; Faundes, V.; Morales, P.; Pugin, Á.; Curotto, B.; Soto, P.; Peña, M.I.; Salas, I.; Alliende, M.A. FMR1 Gene Mutations in Patients with Fragile X Syndrome and Obligate Carriers: 30 Years of Experience in Chile. *Genet Res (Camb)* **2016**, *98*, e11, doi:10.1017/S0016672316000082.
41. Pozo-Palacios, J.; Llamos-Paneque, A.; Rivas, C.; Onofre, E.; López-Cáceres, A.; Villareal, J. Experiences of the Molecular Diagnosis of Fragile X Syndrome in Ecuador. *Front Psychiatry* **2021**, *12*, 716311, doi:10.3389/fpsyt.2021.716311.
42. Mitchell, R.J.; Holden, J.J.A.; Zhang, C.; Curlis, Y.; Slater, H.R.; Burgess, T.; Kirkby, K.C.; Carmichael, A.; Heading, K.D.; Loesch, D.Z. FMR1 Alleles in Tasmania: A Screening Study of the Special Educational Needs Population. *Clin Genet* **2005**, *67*, 38–46, doi:10.1111/j.1399-0004.2004.00344.x.
43. Meguid, N.; Abdel-Raouf, E.; Daedir, A.; Awady, M. Prevalence of Fragile X Syndrome among School-Age Egyptian Males. *World J Pediatr* **2007**, *3*, 271–275.
44. Goldman, A.; Jenkins, T.; Krause, A. Molecular Evidence That Fragile X Syndrome Occurs in the South African Black Population. *J Med Genet* **1998**, *35*, 878, doi:10.1136/jmg.35.10.878.
45. Essop, F.B.; Krause, A. Diagnostic, Carrier and Prenatal Genetic Testing for Fragile X Syndrome and Other FMR-1-Related Disorders in Johannesburg, South Africa: A 20-Year Review. *S Afr Med J* **2013**, *103*, 994–998, doi:10.7196/samj.7144.
46. Hunter, J.; Rivero-Arias, O.; Angelov, A.; Kim, E.; Fotheringham, I.; Leal, J. Epidemiology of Fragile X Syndrome: A Systematic Review and Meta-Analysis. *Am J Med Genet A* **2014**, *164A*, 1648–1658, doi:10.1002/ajmg.a.36511.
